# The relationship between theory of mind and moral sensitivity among Chinese preschool children: the mediating role of empathy

**DOI:** 10.1186/s40359-024-01600-4

**Published:** 2024-03-01

**Authors:** Jun Du, Yuan Liang, Di Guo, Ying Xiao

**Affiliations:** 1 School of Education, Longdong University, 745000 Qingyang, China; 2 School of Psychology, Shaanxi Normal University, 710062 Xi’an, China; 3 Key Laboratory of Artificial Intelligence and Language Cognitive Neuroscience, Xi’an International Studies University, 710128 Xi’an, China; 4 School of Early Childhood Education, Shaanxi Xueqian Normal University, 710061 Xi’an, Shaanxi China; 5 Center School of Languan Street in Lantian District, 710128 Xi’an, China

**Keywords:** Moral sensitivity, Preschool children, Multigroup mediation analyses, Theory of mind, Empathy

## Abstract

**Background:**

Identifying moral behavior in complex situations is the key ability for children to develop prosocial behavior. The theory of mind (ToM) and empathy provide the cognition and emotional motivation required for the development of moral sensitivity. In this study, we investigated the associations among ToM, empathy, and moral sensitivity and explored the possible differences between Chinese preschool children aged 4 and 5 years.

**Methods:**

One hundred and thirty children completed the unexpected-content and change-of-location tasks as well as questionnaires about empathy and moral sensitivity individually. A one-way analysis of variance and the multi-group mediation SEM were used to examine the associations of the three variables and age differences.

**Results:**

The scores of 5-year-old children in the dimensions of care, fairness, authority, and sanctity and the total score were higher than those of 4-year-old children. Moral sensitivity was positively correlated with both ToM and empathy after we controlled for verbal IQ and gender. Multigroup mediation analyses showed age-based differences in the associations among moral sensitivity, ToM, and empathy. Empathy’s mediation effect was partial among 4-year-old children and complete among 5-year-old children.

**Conclusions:**

These findings contribute to understanding the cognitive and emotional factors in the formation of children’s moral sensitivity. They also point to a promising approach to promoting the development of moral sensitivity and evidence for educators to understand the process of children’s socialization.

## Introduction

Moral cognition is the basis for the formation of prosocial behavior [1] and a prerequisite for high-quality interpersonal interactions [2]. Situated at the onset of moral cognition [3, 4], moral sensitivity is significantly influenced by theory of mind (ToM) [5] and empathy [6, 7], particularly in preschool children. According to a hierarchical model of social cognition [8], ToM and empathy not only affect moral sensitivity independently, but also work together in moral sensitivity. However, the pathways by which ToM and empathy are connected with the emerging development of moral sensitivity remain unclear. Moreover, although the development of age-related and individual variations in moral sensitivity has been widely documented [9–11], little is known about the influence of ToM and empathy beyond those differences accounted for by maturation. The ages of 4 to 5 are a critically important period for moral development [12, 13]. Therefore, in the current study, we explore the possible influence of ToM and empathy on moral sensitivity and the stability among children aged 4 and 5. Elucidating the formation mechanism of moral sensitivity in preschool children could provide evidence of the dynamic role of the cognitive and emotional aspects played in the formation of children’s moral development and socialization.

### Moral sensitivity

Moral sensitivity refers to the ability to identify moral behavior in complex situations. This includes understanding others’ reactions and emotions, recognizing how certain behaviors influence well-being and forging informed responses and inferences based on others’ actions [14]. According to the moral foundation theory [3], a novel morality system inspired by evolutionary psychology and multiculturalism [15], moral behavior aligns with four theoretical hypotheses (morality is an innate response mechanism, morality is culturally malleable, moral judgements are perceptual first and reasoned second, and moral content is pluralistic) and comprises five components (care/harm, fairness/cheating, loyalty/betrayal, authority/subversion, and sanctity/degradation) [16]. Moral sensitivity is one of the psychological components of moral behavior [12, 13], and it also has five components: (a) care/harm, which indicates that human beings have to have a kindheartedness and compassion to care for and protect others from harm and have gradually developed a certain altruism; (b) fairness/cheating, which refers to the quality of punishing the cheater and rewarding the fair distributor in the exchange of value with unrelated persons or groups to ensure that neither party’s interests are neglected and that the fruits of co-operation are not plundered; (c) loyalty/betrayal, which means that individuals must be loyal to their in-group to enhance group cohesion and gain more resources and benefits in the competitive environment of different groups; (d) authority/subversion, which refers to the notion that different classes have different powers and obligations in the group of hierarchical structure; and (e) sanctity/degradation, which refers to the moral imperative that individuals keep clean, pursue spiritual sublimation, stay away from unclean people and things, and avoid dirt and filth. We intend to examine moral sensitivity from the above five components.

Additionally, there were great age-based differences in the performance of moral sensitivity among preschool children. Specifically, children become sensitive to authority at the age of 2 but are not able to understand its criterion until the age of 3 [17]. At the age of 4, children begin to express moral judgments and reasoning and protest moral violations, and they decreasingly rely on interpersonal force [17, 18]. They also show a preference for a morally good agent (helping, fair, or comforting) over a morally bad one (hindering, unfair, or hurting) [1, 19]. Furthermore, children aged 3 to 5 can judge the quality of friendships by loyalty, are more attracted to faithful people, and rate loyal behavior positively and disloyal behavior negatively [10]. For the dimension of sanctity, children over the age of 6 exhibit basic cognitive understanding of moral disgust, whereas this cognitive ability tends to stabilize in children around the age of 10 [11]. Thus, the age at which children acquire these moral sensitivity abilities is important to their long-term development, while the influence of age-of-acquisition is still not well understood.

### The relationship between theory of mind and moral sensitivity

ToM refers to the ability to understand that others have mental states that can differ from one’s own [20], and it develops rapidly in preschool children [21]. ToM ensures that children perceive and interpret the needs and feelings of others correctly from cognitive (the ability to make inferences about others’ desires, beliefs, etc.) and emotional (the ability to make inferences about others’ emotions based on facial expressions, body movements, etc.) perspectives [22, 23]. ToM is vital for preschool children in developing moral sensitivity [24] and making moral decisions [25–28]. Iindividuals with more advanced ToM can recognize others’ mental states more accurately and are more able to consider problems from others’ perspectives [29] and then make an appropriate moral judgement [30]. Specifically, ToM could predict the frequency of sharing behaviours among children 3 to 9 years old [31]. In addition, ToM can promote direct reciprocal behaviour of children. Children who performed false-belief tasks well were more likely to make generous offers in a dictator game^1^ following a fair offer made by their partner in a proceeding ultimatum game^2^ [32]. Moreover, ToM positively predicted young children’s moral performance in the care and fairness dimensions, which were prerequisites for moral behaviours [32]. Then we will speculate that ToM also affects moral sensitivity. However, although ToM could ensure that children correctly perceive and interpret others’ needs and feelings, as well as discern possible interpersonal interactions, it does not ensure that children will utilize the information they gather in a beneficial manner. Emotions help people monitor others’ mental activity and exhibit appropriate behaviours by evaluating the nature of mental states and events [33]. In other words, while ToM provides the understanding, it is the emotional response that ultimately guides the behavior.

### The relationship between empathy and moral sensitivity

Empathy is one’s ability to share and understand the internal mental states of others [34]. It plays a very significance role in the moral perception of social events [35]. Generally, empathy includes cognitive empathy and emotional empathy [36, 37]. Cognitive empathy is defined as the ability to intentionally adopt the other person’s point of view. Emotional empathy refers to the individual being infected by others’ emotions to produce alternative emotions and exhibit the same emotional response. Empathy and moral sensitivity have a complex relationship [7, 33]. According to the nested model, empathy can match moral perceptions with actions in a certain social situation [38] and provides the emotional motivation for the development of moral sensitivity in children. Specifically, children with higher empathy were more likely to perceive sad faces (as opposed to neutral or happy faces), whereas those with lower empathy showed more external behavioral problems and were usually unresponsive or very indifferent to others in pain and distress [39]. Empathy without obvious personal interests contributes to the development of moral sensitivity [7]. The positive association between moral sensitivity and moral emotions in preschoolers influences children’s understanding of emotional responses to moral norms [2].

On the differences and connections between ToM and empathy, first, although ToM could ensure that children perceive and interpret others’ needs and feelings and identify the ways others can interact correctly, it cannot guarantee that children use the information they receive in a positive way. With empathy, people monitor the mental states and perform appropriate behaviors by assessing the nature of mental states and events [35]. Empathy is based on ToM. For an individual to perceive and understand others’ emotions, they must first possess the ability to recognize emotional cues in others and infer their mental states [40, 41]. Second, the emotional component of ToM is closely related to cognitive empathy, but it focuses on different aspects: cognitive empathy emphasizes understanding others’ emotional experiences in specific situations whereas the emotional element of ToM includes the cognitive component, focusing on inferring and predicting others’ emotions after understanding their desires and beliefs. It is an extension of understanding desires and beliefs [42].

### The possible mediation role of empathy in the association between theory of mind and moral sensitivity

According to a hierarchical model of social cognition [8], understanding others’ mental states and behavior could be described from a multilevel model of hierarchical structure. A higher level indicates broader and more abstract categories of functioning whereas a lower one indicates the application of these functions in concrete contexts dictated by certain stimuli and task formats. Specifically, there were three groups in the higher level of our model: (a) primarily cognitive processes that come into play when mentalizing requires self-driven cognition decoupled from the tangible world; (b) predominantly affective processes that are active when we perceive emotions in others based on shared emotional, motor, and somatosensory representations; and (c) combined processes that simultaneously engage both cognitive and affective functionalities.

From this, it can be seen that, on one hand, ToM and empathy influence moral sensitivity independently, as we discuss in Sect. 1.2 and 1.3. On the other hand, ToM and empathy work together to influence moral sensitivity. Therefore, empathy would be affected by children’s ability to mentally characterise emotional events and others’ minds [43]. The developments in ToM have substantial implications for children’s development of empathy. For example, 3- and 4-year-olds understand that desires and beliefs may underlie certain emotional reactions (such as happiness, sadness, and surprise) [21]. By around the age of 4, children can utilize their developing ToM to demonstrate an emerging understanding that others may hold false beliefs [44]. Furthermore, behaviors concerning others’ thoughts and experiences are part of generating empathy [45]. Understanding the contribution of others’ mental state is conducive to the improvement of empathy. Therefore, we can speculate that empathy mediates the relationship between ToM and moral sensitivity. That is, more advanced ToM may help an individual to be more empathic by understanding others’feelings more accurately, thus promoting moral sensitivity.

In addition, given that children’s ability to understand others’ reactions develops significantly between the ages of 2 and 5, with a rapid development observed by the age of 4 or 5 [46], and that empathy’s effect on moral sensitivity becomes more prominent with age [7, 47], our main concern here was whether the mediation translation differs from across-age change improvement in the accuracy of non-symbolic numerical representation.

### The current study

**Fig. 1:**
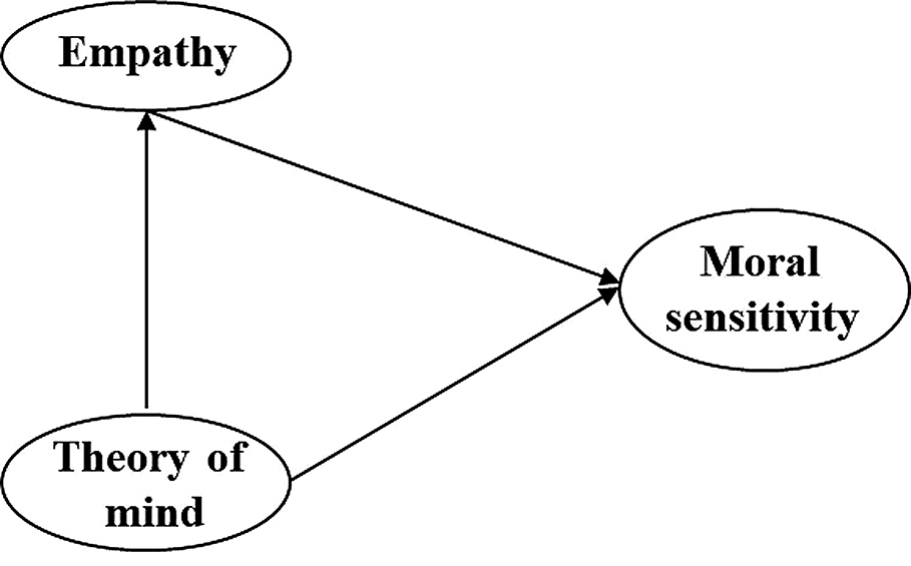
Structural diagram of the hypotheses

Altogether, we aimed to investigate the associations among ToM, empathy, and moral sensitivity and to explore the mediation effect of empathy on ToM and moral sensitivity as well as the differences in mediated pathways between 4-year-old children and 5-year-old children. To address these questions, we assessed 4- and 5-year-old children’s performance on classical ToM tasks, an empathy test, and a moral sensitivity questionnaire during their preschool years. We used a structural equation model and a multi-group mediation model to do so. We hypothesized that empathy plays a mediating role in the relationship between ToM and moral sensitivity (as Fig. 1 shows), and that age-based differences exist in the mediating effect.

## Method

### Participants

We worked with 132 children aged 4- to 5-year-old (*M* ± *SD* = 4.58 ± 0.50; 57 for 4-year-olds and 75 for 5-year-olds) 3from a general kindergarten in Qingyang City, Gansu Province, China. All the participants were recruited from a Chinese general kindergarten in Grade 2 and Grade 3. In China, kindergarten has three grade levels, Grade 1 (3- to 4-year-old children), Grade 2 (4- to 5-year-old children), and Grade 3 (5- to 6-year-old children). The age range of 4-year-old children was from 45 to 50 months, and that of 5-year-old children was 57 to 62 months. To be eligible for inclusion, children had to be native Mandarin-speaking and without a history of neurological, psychiatric, or brain injury problems, and all had normal or corrected-to-normal vision. Additionally, because all the measurements were administered verbally, all of the participants had to have a normal verbal IQ. Two 4-year-old children with low verbal IQ (below the 25th percentile) were excluded. Most of the participants came from low- and middle-income families. Children were instructed to complete tasks regarding ToM, an empathy test, and a moral sensitivity questionnaire individually. The study was approved by the Ethics Committee on Human Experimentation for the School of Psychology, Shaanxi Normal University. Parents of all children provided written informed consent prior to the study. All the children received a small gift (e.g., pencil, stickers) for their participation.

Participants’ verbal IQ was measured as a control variable by using the Chinese version of the Wechsler Preschool and Primary Scale of Intelligence, Fourth Edition (WPPSI-IV(CN)), as revised by ZHANG Houcan (2014) [49]. Verbal IQ includes Information and Similarities. The Information section contains 29 items. Items 1 to 4 require children to choose the best answer from four pictures, and items 5 to 29 required them to answer a wide range of questions. The two tests would be terminated if participants answered four consecutive questions incorrectly. The similarities section contains 23 items. Items 1 to 4 require children to look at two pictures of similar items, and then to choose a picture that belongs to this category. Items 5 to 29 ask children to describe how the two common characteristics or concepts read by the experimenter were similar. The test was discontinued after 3 consecutive incorrect responses. All the participants’ verbal IQs were normal. The mean score and standard deviation were 25.3 and 2.38, respectively.

### Measures

#### Tasks regarding theory of mind

The tasks regarding ToM were primarily unexpected-content and change-of-location tasks, which were adapted from the classic paradigm developed by Wimmer and Perner (1983) developed [50].

##### Unexpected-contents tasks

The instructor showed a participant a toothpaste box containing a pen, stating, “This is a toothpaste box. What do you think is inside?” The participant was then asked to open the box, remove the pen, and then return the pen back to the box, restoring its initial state. Following this, the instructor asked, “What is now inside the toothpaste box?” If the child responded inaccurately, they were then gently guided toward the correct answer.

The following were the questions pertaining to representational change and false belief: “Before you opened the toothpaste box, what did you think was inside, a pen or toothpaste?” and “If another child came over, please guess what the child would think is inside the box, a pen or toothpaste.” The corrected answer for both questions was ‘toothpaste’.

Participants got 2 points for correctly answering both questions, 1 point for one correct answer, and 0 point for incorrect answers. Cronbach’s α was 0.69. The correlation of the two measurements was 0.39 (*p* < 0.001).

##### Change-of-location tasks

The instructor told the participant a story using a cartoon picture featuring Peppa Pig and then asked the participant corresponding questions. The story was as follows: “Look, this is Peppa. She is playing with her toy plane in her room. After a while, she puts the toy plane on her bed and goes outside to play. Soon, her mother comes into her room, spots the toy plane on the bed, and, thinking it creates a mess, places it inside the cupboard. ”

Two control questions followed the narration. The first question was “Where did Peppa initially place her toy plane?” (The correct answer is ‘the bed’). The second question was “Where is the toy plane now?” (The correct answer is ‘in the cupboard’). If the participant answered incorrectly, they were guided to the right answer.

There were also two questions. The first was “After a while, Peppa returns and wants to play with the toy plane. Where would Peppa imagine the plane is?” The second question was “Where would Peppa go to look for her toy plane?” The correct answer to both questions was ‘The bed’.

The control questions were used to determine whether participants were able to understand the test tasks. Once a participant correctly answered the control questions, the test questions were posed. If the control questions were not answered accurately, the test procedure was terminated. The scoring rules were as above. The Cronbach’s α was 0.75. The correlation of the two measurements was 0.38 (*p* < 0.001).

#### Empathy test

Empathy was measured using the Feshbach Affective Situations Test for Empathy (FASTE) [51–53], and an empathy continuum instrument [54]. The former was used to assess affective empathy, which contains 4 emotional situations (sadness, happiness, anger, and fear), and the latter was used to measure cognitive empathy. Children completed the empathy test in two steps. The first step was to complete the task in the FASTE, and the second step was to complete the questions in the empathy continuum instrument.

The children were first shown an image depicting a situational story in group settings. They were then prompted to express their emotional state, either verbally or by choosing an emotion card that accurately represented their feelings. After comprehending the specific stories, the participants articulated their emotional states using corresponding pictures. Take the sad situational story as an example: “Peppa had a pet dog who was also her best friend. The dog followed Peppa everywhere she went. Regrettably, the dog ran away and Peppa couldn’t find it ever again. She lost her puppy forever.”

Following the completion of this task of reporting emotional states, the children were required to answer two questions posed in the empathy continuum instrument. The first question was “What do you think Peppa is feeling in the story?” The second question was “How does listening to this story make you feel?” The participants chose from the following four emotions: (a) happy, (b) sad, (c) angry, and (d) scared.

Children who could correctly identify the main character’s (Peppa’s) emotion in the story (a demonstration of cognitive empathy) would get 1 point. They could gain another point for reporting the same emotion as the main character in the story (showing affective empathy). Those who accurately completed both tasks got 2 points. The total scores for the four emotions range from 0 to 8 points.

#### Questionnaire for moral sensitivity

The Situational Questionnaire for Moral Sensitivity of children was developed by Du (2020) [55], based on the Moral Foundation Vignettes [56]. The questionnaire includes five dimensions (care, fairness, loyalty, authority, and sanctity) and 28 items in total. The Cronbach’s α for the five dimensions were 0.91, 0.78, 0.76, 0.82, and 0.78, respectively. Specifically, care involved children’s moral awareness and evaluation of behaviours situations of emotions, bodies, and animal injury. A sample item is “A child punched another classmate.” Fairness involved children’s moral perceptions and evaluations of unfair and deceptive behaviour situations. An item is “One child gives 2 gifts to Xiao Hong and to 3 gifts to Xiao Ming in the classroom.” Loyalty involved children’s perceptions and evaluations in disloyalty and betrayal situations. An item is “One child said the preferred class was the other class.” Authority involved children’s moral perceptions and evaluations of situations in which they do not respect authority. An item is “One child did not listen to the teacher’s guidance.” Sanctity involved children’s moral awareness and evaluation of staying clean and holy. An item is “One student used someone else’s toothbrush to brush their teeth.” Overall, the Cronbach’s α was 0.96, and the confirmatory factor analysis revealed the questionnaire’s good construct validity (χ^2^/df = 2.16, GFI = 0.94, CFI = 0.97, NFI = 0.95, TLI = 0.98, SRMR = 0.04, RMSEA = 0.05).

**Fig. 2:**
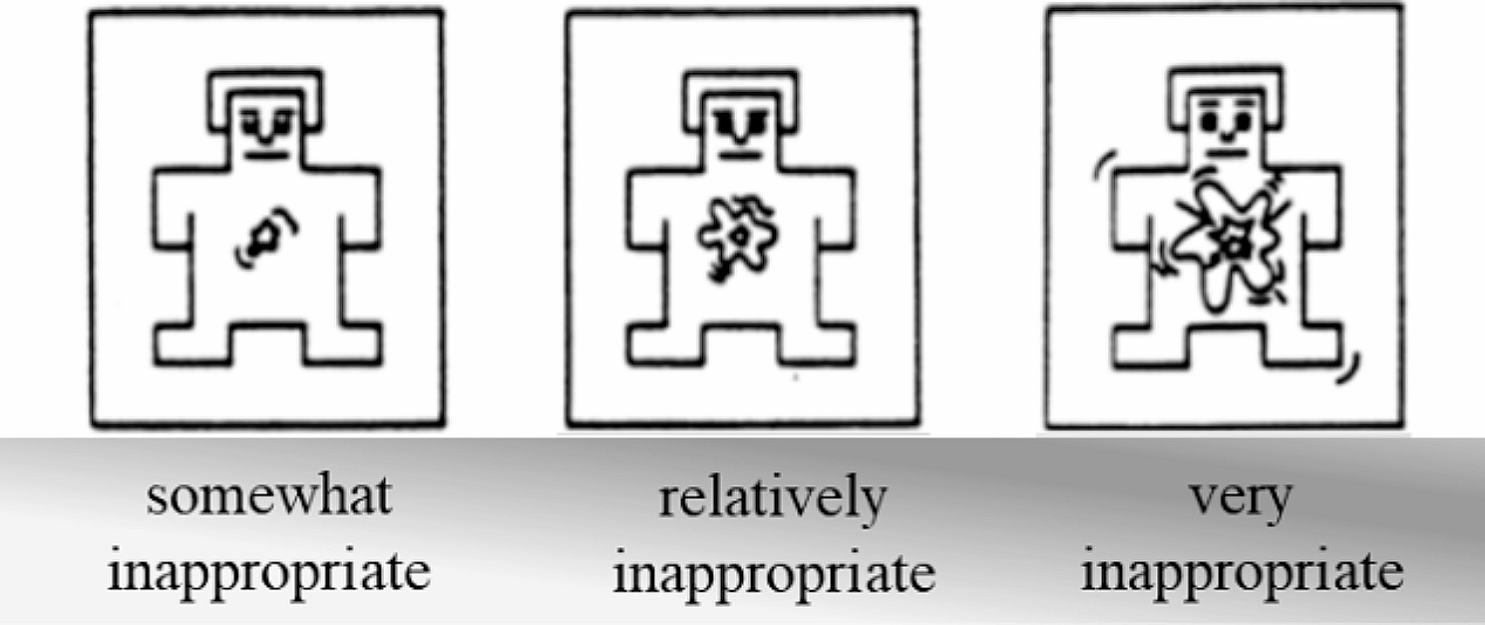
Visual aids assisting children to judge indivuduals’ behavior in moral sensitivity situations

The questionnaire included two-step questions and a four-point scale, asking children to judge the rightness or wrongness of an ethical violation and rate its wrongness. Children were first asked whether a person’s behavior in the scenario was proper. If a child answered “‘yes”, he/she would get 0 points. If the answer was “no,” the second question would be “Is the behavior somewhat inappropriate, relatively inappropriate, or very inappropriate?” (The cartoons in Fig. 2 could help the children understand). If the child could make a choice, he/she would get 1 point for choosing “somewhat inappropriate,” 2 points for “relatively inappropriate,” and 3 points for “very inappropriate.” The score for each dimension is the average of the total score of all items in the dimension, which ranged from 0 to 3. The total score on moral sensitivity is the sum of the scores on all dimensions.

### Procedure

Trained instructors who were undergraduate students majoring in preschool education conducted all the tests individually in a quiet, well-lit kindergarten classroom. Children’s answers and responses were recorded.

### Data analysis plan

First, we presented the descriptive statistics and the correlation coefficients for all the study variables and their dimensions. Second, we examined the age-based differences in moral sensitivity by conducting a one-way analysis of variance. Third, we conducted a multigroup mediation structural equation model to explore whether empathy’s effect on the correlations between ToM and moral sensitivity were different between 4- and 5-year-old children. The purpose of the multigroup analysis was to determine whether the mediation model was equivalent across groups or the parameters were invariant [48]. Specifically, we investigated the significance of direct effects from ToM to empathy (Path a), the path from empathy to moral sensitivity (Path b), and the path from ToM to moral sensitivity (Path c). All pathways were controlled for verbal IQ and gender. Additionally, we conducted the bias-corrected bootstrapping approach to examine empathy’s indirect effects on the association between ToM and moral sensitivity. The bias-corrected bootstrapping approach does not need to satisfy the normality assumption. In addition, the unstandardized bootstrapped 95% confidence intervals (*CI*s) were used to determine the significance of the indirect effects (significant effect is indicated by *CI*s that do not contain zero) [57]. The data analyses were conducted using Mplus 8.0 and SPSS 22.0.

## Results

### Preliminary analyses

Table 1 shows the descriptive statistics and correlations among the measured study variables. As expected, all five dimensions of moral sensitivity were relatively strongly correlated (*r* ranged from 0.56 to 0.87, *p*s < 0.001), regardless of whether IQ, gender, and age were controlled. With the exception of fairness, all other variables were significantly associated with the unexpected content. In contrast, the two dimensions (e.g., care and sanctity) were significantly associated with the unexpected location, but the other three dimensions (e.g., fairness, loyalty, and authority) were not. After we controlled for IQ, gender, and age, only the association between the unexpected contents and loyalty remained significant. Table 2 presents the correlations among the total scores of the three variables. Moral sensitivity, ToM, and empathy were significantly correlated with each other, regardless of whether the verbal IQ, gender, and age were controlled. Combining the results in Tables 1 and 2, we can speculate that the correlation coefficients between ToM and moral sensitivity decreased with the addition of control variables whereas the values for empathy and moral sensitivity remained very significant.

**Table 1:** Descriptive statistics and correlations among subdimensions of theory of mind, empathy, and moral sensitivity

Variables	M(SD)	1	2	3	4	5	6	7	8	9
1. unexpected content	0.82(0.80)		*0.23* ^**^	*0.19* ^*^	*0.04*	*0.14*	*0.09*	*0.19* ^*^	*0.16*	*0.16*
2. unexpected location	0.70(0.78)	0.32^***^		*0.11*	*0.02*	*0.02*	*0.03*	*0.09*	*0.02*	*0.11*
3. affective empathy	5.23(1.62)	0.25^**^	0.17^*^		*0.20* ^*^	*0.25* ^**^	*0.03*	*0.16* ^*^	*0.19* ^*^	*0.25* ^**^
4. cognitive empathy	3.25(0.82)	0.01	0.05	0.23^**^		*0.32* ^***^	*0.31* ^***^	*0.26* ^**^	*0.34* ^***^	*0.35* ^***^
5. care	3.25(0.77)	0.22^**^	0.16^*^	0.30^**^	0.34^***^		*0.75* ^***^	*0.66* ^***^	*0.86* ^***^	*0.82* ^***^
6. fairness	3.12(0.90)	0.15	0.11	0.12	0.34^***^	0.76^***^		*0.74* ^***^	*0.64* ^***^	*0.74* ^***^
7. loyalty	2.88(0.91)	0.23^**^	0.14	0.19^*^	0.33^***^	0.68^***^	0.76^***^		*0.56* ^***^	*0.66* ^***^
8. authority	3.34(0.83)	0.24^**^	0.09	0.24^**^	0.28^***^	0.87^***^	0.67^***^	0.58^***^		*0.75* ^***^
9. sanctity	3.20(0.85)	0.24^**^	0.21^*^	0.30^**^	0.35^***^	0.85^***^	0.75^***^	0.67^***^	0.77^***^	

The above the diagonal is the correlation value (italic form) among variables after controlling for IQ, gender, and age, the below is not

*Note*. ^*^p < 0.05, ^**^p < 0.01, ^***^p < 0.001

**Table 2:** Pearson correlations among theory of mind, empathy, and moral sensitivity

Variables	No control variables			Control IQ, gender, and age	
	1	2		1	2
1. moral sensitivity					
2. theory of mind	0.25^**^			0.20^*^	
3. empathy	0.36^***^	0.22^**^		0.32^***^	0.19^*^

*Note*. ^*^p < 0.05, ^**^p < 0.01, ^***^p < 0.001

Considering the possible age-based differences in moral judgments of 4- and 5-year-olds, we examined their differences in the five moral dimensions and relationships among ToM, empathy, and moral sensitivity. Results were presented in Tables 3 and 4. On average, in addition to the loyalty dimension, the scores on the other four dimensions and moral sensitivity were significantly higher for 5-year-olds than for 4-year-olds. Additionally, after we controlled for IQ and gender, the associations among the three variables did not change (*r*s changed from 0.19 to 0.36, *p*s < 0.01), and after we controlled for age, it changed marginally significant (*r*s changed from 0.16 to 0.33, *p*s < 0.10). The above results suggest that the relationships among the three variables were different for 4- and 5-year-old children. Moreover, Cohen (1992) assumed that the coefficient of the Pearson correlation itself represents the effect size [58]. Accordingly, the criteria for small, medium, and large effect sizes are *ρ* = 0.1, *ρ* = 0.3, and *ρ* = 0.5, respectively. All correlation coefficients in this study were medium effects, and all the statistical test powers were above 0.99. These provided the necessary prerequisites for constructing the SEM model.

**Table 3:** Pearson correlations among theory of mind, empathy, and moral sensitivity

	No control		Control IQ		Control gender		Control age	
Variables	1	2	1	2	1	2	1	2
1. moral sensitivity								
2. theory of mind	0.25^**^		0.22^**^		0.25^**^		0.16^†^	
3. empathy	0.36^***^	0.22^**^	0.34^***^	0.19^*^	0.36^***^	0.22^**^	0.33^***^	0.16^†^

*Note*. ^†^*p* < 0.10, ^*^p < 0.05, ^**^p < 0.01, ^***^p < 0.001

**Table 4:** Differences in the moral sensitivity between children aged 4 and 5(*M*(*SD*))

Age (years)	Care	Fairness	Loyalty	Authority	Sanctity	Moral sensitivity
4(*n* = 55)	3.01(0.93)	2.91(1.05)	1.73(1.03)	3.06(0.98)	2.91(0.98)	2.92(0.90)
5(*n* = 75)	3.43(0.57)	3.27(0.73)	2.98(0.80)	3.55(0.63)	3.41(0.66)	3.33(0.57)
*F*	10.47^**^	5.29^**^	2.51	11.91^***^	12.21^***^	9.86^**^
η_p_^2^	0.08	0.04	0.02	0.09	0.09	0.07

*Note*. ^**^p < 0.01, ^***^p < 0.001

### Multigroup mediation analyses

**Fig. 3:**
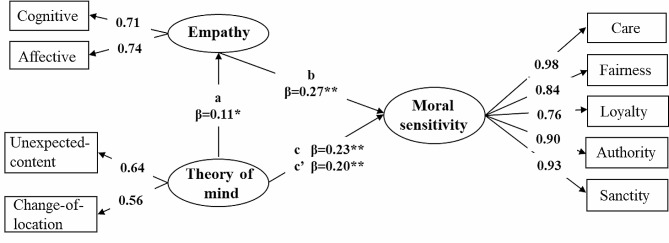
The mediation model of empathy for 4-year-old children. *Note*: Standardized parameter estimates are reported after controlling for gender and verbal IQ. ^*^p < 0.05, ^**^p < 0.01

**Fig. 4:**
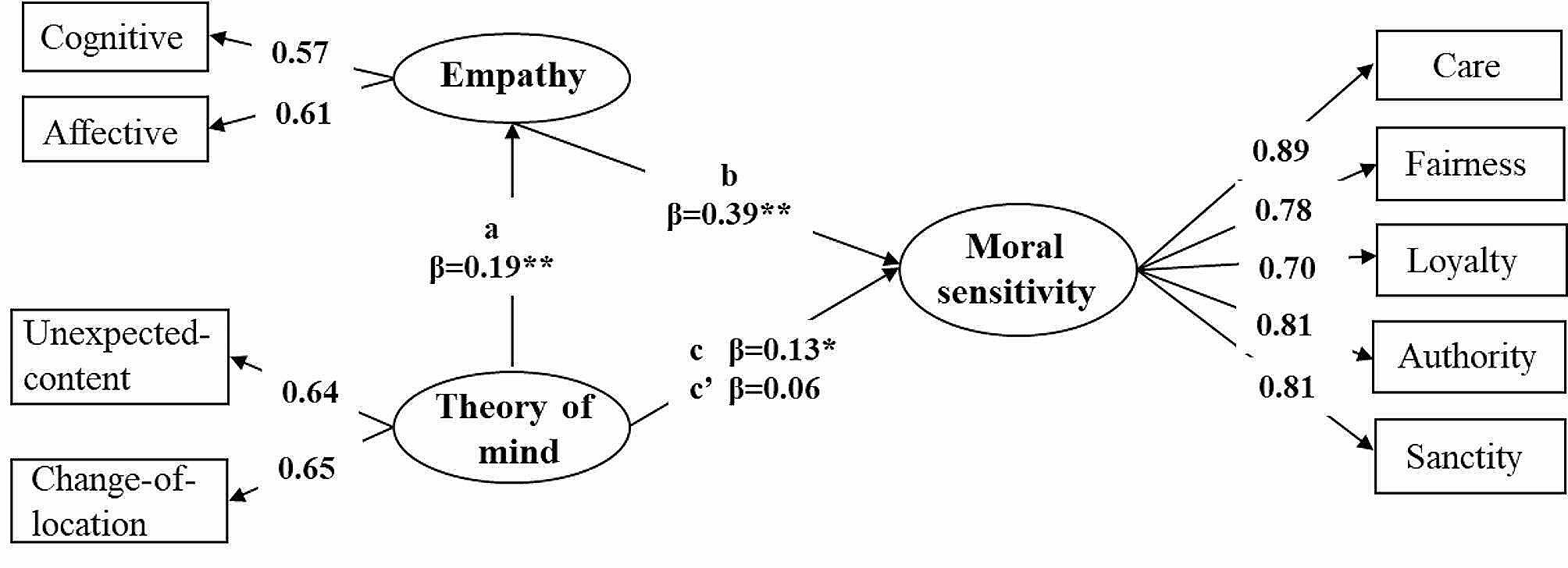
The mediation model of empathy for 5-year-old children. *Note*: Standardized parameter estimates are reported after controlling for gender and verbal IQ. ^**^p < 0.01, ^***^p < 0.001

Considering the significant differences between moral sensitivity and correlations among the three variables in 4- and 5-year-old children, we conducted a multigroup mediation model of age differences. The model fit the data well (χ^2^/df = 4.00, RMSEA = 0.04, TLI = 0.97, CFI = 0.99). Specifically, for 4-year-old children, ToM only influenced moral sensitivity directly but also affected moral sensitivity through empathy indirectly. For 5-year-old children, ToM not only affected moral sensitivity, but when empathy was added, the influence path was no longer significant. Table 5 presents the standardized and unstandardized coefficients for all the directional paths among ToM, empathy, and moral sensitivity in this model (with standard errors) for 4- and 5-year-old children. Figure 3 (4-year-olds) and Fig. 4 (5-year-olds) depict the statistically significant directional paths in the model with standardized βs.

**Table 5:** Mediation analysis: direct and indirect effects of ToM on moral sensitivity

	Total effect	Direct effect	Indirect effect: The mediation effect of empathy		
4-year-old	c	c’	a	b	a×b
	β [95%CI]	β [95%CI]	β [95%CI]	β [95%CI]	β [95%CI]
Theory of mind	0.23	0.20	0.11	0.27	0.03
/Moral sensitivity	[0.05, 0.35]	[0.12, 0.26]	[0.03, 0.55]	[0.01, 0.23]	[0.00, 0.04]
5-year-old	c	c’	a	b	a×b
	β [95%CI]	β [95%CI]	β [95%CI]	β [95%CI]	β [95%CI]
Theory of mind	0.13	0.06	0.19	0.39	0.07
/Moral sensitivity	[0.09, 0.27]	[-0.12, 0.16]	[0.01, 0.59]	[0.02, 0.19]	[0.00, 0.11]

Note. CI = confidence interval. β refers to the standardized parameter estimates

## Discussion

We aimed to explore the age-based differences in the association among ToM, empathy, and moral sensitivity among 4- and 5-year-old Chinese preschool children. The 5-year-olds scored higher on all the dimensions and total moral sensitivity than the 4-year-olds. Moral sensitivity was positively correlated with ToM and empathy after we controlled for verbal IQ and gender. Multigroup mediation analyses showed age-based differences in the associations among ToM, empathy, and moral sensitivity. For 4-year-old children, empathy plays a partial mediating role between ToM and moral sensitivity whereas for 5-year-old children, empathy fully mediates the relationship between ToM and moral sensitivity.

Our findings elucidate how ToM impacts moral sensitivity and provide empirical evidence of the hierarchical model of social cognition [8]. This model showed three ways that cognition and emotion affect moral sensitivity, primarily cognitive processes, predominantly affective processes, and combined processes that simultaneously engage cognitive and affective functionalities. ToM provides the cognitive reasoning path of moral sensitivity [20], and empathy provides an emotional understanding path of it [24].

First, for the primarily cognitive processes, we found that ToM predicted moral sensitivity significantly. According to the theory of moral cognition development [59, 60], moral development is a continuous process characterized by stages and sequences, which is based on cognitive development. Four years old is a critical age milestone for a significant shift in children’s ToM. The acquisition of ToM allows children to gradually move away from understanding the essence of things solely from their perspective and recognize that others may have beliefs and behaviors different from theirs [61]. This in turn affects their moral sensitivity.

Second, empathy can certainly affect and contribute to moral behavior [28, 30, 33, 62]. It predicts social skills, emotional stress, and behavioral problems in young children [58]. Empathy is a construct comprising emotional and motivational components, which interact and operate in a parallel way [33]. Empathy-related processes are thought to motivate prosocial behavior (e.g., sharing, comforting, and helping) and caring for others; initiate emotional sharing, empathic concern, and affective perspective taking; inhibit aggression; and provide the foundation for care-based morality [33]. Specifically, individuals with a high level of empathy could perceive others’ feelings, explain things from others’ perspectives, and strengthen their communication with others. In contrast, individuals with weak empathy were unable to understand others’ thoughts, could not interact with others normally, and even became violent [29].

Third, our study reveals the age-based differences in empathy’s mediation effect on the association between ToM and moral sensitivity. The multigroup mediation model revealed that empathy partially mediates the relationship between ToM and moral sensitivity for 4-year-old children, and it emerged as a complete mediator in the relation between these two variables for five-year-old children. On one hand, 5-year-old children performed better on all the dimensions of moral sensitivity except for loyalty than 4-year-old children. The evidence showed that with the improvement of cognitive level, care sensitivity [63], fairness sensitivity [64], authority sensitivity [17, 65], and sanctity sensitivity [66] all gradually improved. Not until children are over 4 years do they start to show loyalty awareness. Children aged 4 and 5 prefer members of their own team [67] and are more willing to strive for loyalty to their team [68]. Five years old is a critical stage of cognitive development [69]. Most children gain a cognitive understanding of “false beliefs,” and their cognitive empathy develops more quickly than their emotional empathy [70]. On the other hand, ToM predicted empathy, which in turn predicted moral sensitivity. That is, a more advanced ToM facilitates stronger empathy, which in turn brings about stronger moral sensitivity. Moreover, the process by which children think about others’ thoughts helps generate empathy. Additionally, emotional information can bypass the conceptual-reasoning system and help express moral sensitivity. This result paved the way to a better theoretical framework for further investigations.

### Limitations and implications

This study revealed the mediating role of empathy in the influence of ToM on moral sensitivity among Chinese preschool children. However, the study has three possible limitations. The first is the very limited number and dimensions of ToM tasks, which may decrease the test validity. Additionally, the small sample size affects the model’s stability and the generalizability. Secondly, the experimental context limits the generalizability that would qualify children’s understanding of the ToM tasks in various cultural situations. Researchers should assess the understanding bias caused by cultural differences for cross-validation purposes. In addition, the cross-sectional nature of our data also makes it impossible to infer causal relationships among all the three variables. We therefore described our results in terms of associations and predictive effects. Researchers should conduct a longitudinal study to further explore the developmental characteristics of the causal relationships among these variables. Moreover, we cannot include all mediation variables to explain the link between ToM and moral sensitivity. For instance, executive function predicted later variation in false-belief understanding or ToM for children aged 3 to 6 [71] and predicted lying behaviours (a performance of moral violations) significantly for preschool children [72]. Emotional understanding also played a key role in the development of appropriate moral judgment among preschool children [62] and predicted children’s moral reasoning concurrently and prospectively [2, 9]. Researchers should examine these factors’ possible explanatory effect on the associations between ToM and moral sensitivity, it may be more instructive to cultivate preschool children’s prosocial behavior.

## Data Availability

The datasets during and/or analyzed during the current study available from the corresponding author on reasonable request.

## Footnotes

1 The dictator game is mainly used to examine children’s and adults’ altruistic behavior in diverse cultures because of its simplicity, uniform procedure and tightly controlled situation. In a typical dictator game, the proposer (or dictator) is given an amount of money or some valued resource and asked to divide it between himself/herself and the recipient (or receiver).

2 The ultimatum game is mainly used to investigate the fairness in economic decision-making. In the Ultimatum Game, a sum of money is split between a proposer and a responder. The proposer decides how this money should be split between the two. The responder decides whether he or she will either accept or reject this offer. If the responder accepts the offer, the amount of money is split as agreed. The rational strategy is for the proposers to offer the smallest possible positive share and for the responder to accept in turn.

3 Regarding the sample size in our study, in a simulation multi-group analysis study [48], the minimum sample size was 50. We used the G*power 3.1 software to calculate the sample size. When power = 0.80, effect size ≈ 0.15, the minimum sample size was 68. Therefore, a sample of 75 5-year-olds was appropriate, and the sample of 57 4-year-olds barely met the criteria.
